# When all is unequal, the rich get dominant: Inequality leads to expectations of dominant leadership among those high in SES

**DOI:** 10.1371/journal.pone.0321138

**Published:** 2025-04-09

**Authors:** Anita Schmalor, Eric J. Mercadante, Jessica L. Tracy, Steven J. Heine

**Affiliations:** 1 University of British Columbia, Vancouver, Canada; 2 New York University, Stern School of Business, New York, New York, United States of America; Max Planck Institute for Evolutionary Anthropology, GERMANY

## Abstract

People of higher SES have been found to behave more dominantly than people of lower SES. We tested the hypothesis that this difference is exacerbated under conditions of high economic inequality, when the income/wealth difference between those of low and high SES becomes greater. Across four studies (N =  2,739), using both experiments that manipulate perceived inequality (Studies 1a, 1b, and 3) and a correlational study that measures perceived inequality (Study 2), we find evidence that people expect others and themselves to become more dominant if they are of high as opposed to low SES, and this difference is most extreme when economic inequality is perceived to be high.

## Introduction

Humans live in social hierarchies in which their income, wealth, and prestige defines their socioeconomic status (SES). People’s SES, in turn, has profound effects on their psychology. For example, studies in industrialized, individualistic societies have found that those with higher SES tend to have lower levels of emotional intelligence [1–6], greater feelings of entitlement [7], and a more independent self-construal [8]. These outcomes are thought to emerge from individuals of higher SES in these contexts being less dependent on others to help them achieve their desires and needs, which leads to a greater psychological focus on the self and self-relevant outcomes rather than on others and the consequences of one’s behavior on others. In this vein, we expect that the psychological impact of SES also influences the strategies that people use to rise and maintain their position within the social hierarchy.

Importantly, however, the psychological effects of SES in any society must be considered alongside that society’s level of economic inequality. Economic inequality and SES are distinct but related concepts. In a society with perfect equality of income/wealth, there would be no differences in SES/social class (if they arose solely from income/wealth). In other words, any differences in the SES among members of a society necessitate some amount of inequality, but the extent of inequality—and thus the distinction between those who hold low and high SES positions—varies from society to society. Inequality changes the wealth (and/or status) gap between people of low and high SES in a society. When inequality is high, people of high SES have an even greater proportion of the overall resources than they do when inequality is low. Thus, as economic inequality increases, people at the top of the hierarchy accrue more and more power relative to those below them. As a result, the psychological effects of SES in highly unequal societies are likely to be of greater impact than in more equal societies. For example, people of higher SES who perceive high levels of inequality show less emotional intelligence than people of higher SES who perceive low levels of inequality [4]. We hypothesized that the degree of perceived economic inequality in one’s environment would also moderate the effects of SES on dominance behavior.

In this research, we explored whether economic inequality moderates the effect SES has on societal members’ expectations about status striving—and in particular the extent to which people use dominance-based strategies to get ahead. More specifically, dual strategies theory [9] maintains that people can achieve higher status through either the strategies of dominance or prestige. A dominance strategy of status enhancement is characterized by coercion and intimidation. Dominant individuals often take a disproportionately large proportion of available resources for themselves and distribute resources transactionally with their allies in exchange for their loyalty and deference, and they are typically feared by followers [10–14]. A prestige strategy, on the other hand, is characterized by the possession of valued skills and demonstration of expertise, along with the willingness to freely share skills and knowledge with others. Prestige, in contrast to dominance, is granted by others, as followers choose to show deference and respect to prestigious leaders [9,11,15,16].

People who are higher in SES have greater access to valued resources, and wealth can be a reliable proxy for power because it affords the capacity to control one’s own outcomes and exert influence over others’ outcomes [e.g., 16,17]. A dominance strategy is likely to be more successful for individuals who have more access to wealth and power, and is therefore more appealing. Those who hold a disproportionate amount of wealth are more likely to effectively employ a dominance strategy (e.g., threatening and intimidating others) even if other people don’t like it – high SES individuals can claim power and others may lack the resources to stop them. Supporting this expectation, people of higher SES are more likely to use dominant tactics to get their way [16].

The tendency to use a dominant and/or prestige strategy is, in part, a stable personality trait, yet people can employ these strategies flexibly in response to contextual factors [e.g., 18]. Both strategies likely have trade-offs for the individual employing them [13]. For example, a dominance strategy offers the opportunity to maximize the amount of resources an individual can accrue, but, if unsuccessful, bears the risk of ostracism or other forms of punishment. Conversely, a prestige strategy offers the opportunity to influence the group without any need to control or coerce but might require an individual to spend considerable time mastering skills, and, if unsuccessful, could entail a loss of resources that could have gone elsewhere. One question that arises from this analysis is whether higher SES invariably leads people to adopt more dominant strategies or whether contextual factors might moderate this relationship.

We argue that high inequality within a given social group might lead high SES individuals to behave more dominantly toward low SES individuals, specifically, because the transfer of a group’s resources to the top creates an economic ecology where dominance becomes increasingly less risky, as those at the bottom have less access to valuable resources, and hence, less ability to punish dominant strategists. In these contexts, people of high SES should be more tempted to maintain their power over lower SES individuals through the use of dominance-based strategies. While it is definitional that people of high SES have greater access to valued resources relative to their lower SES counterparts, this relation is exacerbated under conditions in which there is greater economic inequality. In contrast, when inequality is low, people at the top have a smaller proportion of the overall wealth, so their chances of successfully employing a dominance strategy should decrease. Thus, in more equal contexts the benefits of employing a dominance strategy may no longer outweigh its costs [see also 18]. Furthermore, in contexts with greater inequality, the perceived risk of status loss among high SES individuals might be greater because the gap between high and low SES is larger, by definition. This might lead high SES individuals to become more willing to engage in dominance-based strategies that seem likely to succeed because they are willing to do whatever it takes to preserve their status. Crucially, these arguments apply to interactions between high and low SES individuals because they are based on the exacerbated differences between these groups in high inequality contexts; interactions between individuals of the same SES are unlikely to be influenced by inequality in the same way due to equivalent resource-richness between parties.

Interestingly, one consequence of dominant individuals occupying high SES within a group might be an increase in inequality over time because dominant individuals are unlikely to altruistically share their resources [10,19], suggesting a possible feedback loop between greater dominance and greater inequality over time. In the present research, we focus on contexts with a given level of inequality and explore the downstream consequences for individuals’ intentions to engage in dominant or prestigious behavior. Thus, we address only one potential causal direction between these two constructs.

Importantly, given that we expect having high SES in a high inequality context helps make dominance-based strategies more effective and insulates dominant individuals from potential backlash, these arguments apply primarily to individuals who are willing and able to engage in dominance-based strategies. This may be because individual differences predispose certain individuals to prefer dominance-based strategies in pursuit of social rank [e.g., antagonism; 10], or because one is willing to engage in whatever strategy they believe will be effective. However, individuals who are averse to using dominance-based strategies for reasons unrelated to the perceived likelihood of success of those strategies [e.g., highly agreeable individuals; 10] should remain averse to these strategies even when occupying high SES in a high inequality context. If this is the case, then we would expect to observe an overall increase in dominance among high SES individuals in high inequality contexts, but this is not because every wealthy person is more motivated to engage in dominance-based strategies to the same degree than they would be in a context with lower inequality.

As a result, a strong test of these hypotheses requires that we sample from a population where at least a substantial portion of individuals have some motivation or predisposition to engage in dominance-based strategies. Dominance-based strategies are more rational under conditions of abundance and resource stability [19,20], suggesting that individuals living in wealthy, industrialized nations are more likely to benefit from dominance, and thus more motivated to utilize these strategies. In our studies, we sample from a population of Americans who voluntarily engage in research studies via crowdsourcing websites, which should provide access to individuals who live in an environment where dominance-based strategies are often rational.

The goal of the present research was twofold. First, we aimed to replicate the previously found positive association between SES and dominance and test whether it holds when dominance is measured using the well-validated and reliable Dominance scale [10]. Second, we aimed to test whether economic inequality moderates this relationship. Specifically, we hypothesized that people of high SES would behave more dominantly in contexts where inequality is high than where it is low. We test these predictions through the lens of social perceptions—how people see their own and others’ social class, inequality, and likelihood of using a dominance strategy. Differences in status and inequality are largely consciously accessible to societal members, and, as a result, individuals may hold expectations that others and themselves will behave more dominantly in the predicted contexts. Holding these conscious expectations would be adaptive, by virtue of affording individuals the ability to adjust the character of their social interactions according to context in a flexible manner.

We therefore focus on people’s perceptions of their own and other people’s social class, and of the amount of inequality in their environment (which we both assess and manipulate). Considerable research shows that subjective SES is an important and often stronger predictor of psychological outcomes than are objective indicators of SES [e.g., 21,22]. Likewise, a growing body of research has found that people’s perceptions of inequality are importantly linked to their psychological experiences. For example, people who perceive more inequality tend to have greater status anxiety, lower emotional intelligence, construe the self as more independent, and are less trusting and cooperative [e.g., 4,23–25].

It may seem surprising that perceptions of inequality have such broad effects, given the finding that people regularly underestimate the actual amount of inequality in their society [e.g., 26–31]. However, objective measures of inequality typically summarize the levels of inequality across a whole country or state, and people may be more attuned to the inequality they perceive in their more immediate local environment [see also 32–34]. More importantly, people rely upon their perceptions to guide their behavior, whether those perceptions are accurate or not [35–36].

To summarize, across four studies (*N* =  2,739) we tested the hypotheses that: (a) people expect others and themselves to behave in more dominant ways when those others or themselves are of high as opposed to low SES, and (b) this tendency is exacerbated under conditions of high perceived inequality.

We also included a measure of prestige in all studies. Prestige and dominance are typically uncorrelated, so change in one strategy is not necessarily related to or accompanied by change in the other [10–11]. Based on past research, we had no clear predictions regarding the relation between SES, inequality, and perceived prestige, so we analyzed effects of prestige in an exploratory manner. We did not find reliable results for prestige; instead, findings were mixed across the four studies.

## Study 1a

In Study 1a, we tested (a) whether individuals would expect others to be more likely to use dominant tactics when they were described as rich as opposed to poor, and (b) whether rich people were seen as most likely to use dominant tactics when living in a highly unequal as opposed to equal society. The materials, data, and analysis code for all studies are available on the OSF at [https://osf.io/jwtd7/?view_only=35da24bbcbf24561b272f6ed0b7acdae]. We conducted all analyses using R [37] and RStudio [38]. We report all manipulations, measures, and exclusions.

### Method

All studies presented in this manuscript were approved by the Behavioral Research Ethics Board and the University of British Columbia (H17-01798). Participants provided written consent before beginning the study procedures.

***Participants.*** We solicited a convenience sample of Americans on Prolific. Estimating a medium effect size of *f* = .25, 80% power, alpha of.05, with two as the number of groups (i.e., the between-level factor, 2 measurements (i.e., the within-level factor), and an estimated correlation of 0.5 among repeated measures, we needed a sample size of about 34 [as calculated in GPower using the F test family and the ANOVA repeated measures, within-between interaction statistical test; 39]. To ensure we obtained a sufficiently large sample to provide stable estimates of the relationships between variables within each condition [40], as well as the power to test for between-condition differences, we collected data from 420 participants. We included two attention check questions in the study and excluded any participants who failed at least one of them (“For this question, please choose the answer on the far right/at the bottom”). We also excluded participants who indicated that their answers shouldn’t be included in response to a question about whether they had taken the study seriously. The final sample size was 368 participants (*M* age =  31.23; 49% female, 50% male, 1% other; 55% Caucasian, 19% Asian, 11% Hispanic, 10% African American, 5% other).

#### Measures and stimuli.

***Economic inequality.*** To manipulate perceptions of inequality, participants were randomly assigned to read a vignette about a society that was either high or low in inequality:

**High Inequality**
Imagine a society where most people have low incomes and own no or little wealth while a few people have extremely high incomes and own almost all of the wealth. In other words, income and wealth inequality are high; the vast majority of people have only a small proportion of the overall income and wealth, while a small minority have most of the income and wealth.
**Low Inequality**
Imagine a society where most people have fairly similar incomes and own a fairly similar amount of wealth. In other words, income and wealth inequality are low; the vast majority of people have pretty similar overall incomes and wealth.

***Dominance & Prestige.*** Participants rated both rich and poor members of the fictional society they had read about using the 17-item Dominance and Prestige scale [10]. After reading the inequality manipulation blurb, all participants were asked to consider what rich and poor people in this society might be like: “In general, what do you think rich [poor] people are like in this society?” Then participants completed the Dominance and Prestige scale [10]. Eight items (e.g., “They enjoy having control over other members of the society”) assess the extent to which others are seen as acting in dominant ways and nine items (e.g., “Other citizens of this society respect and admire them”) assess the extent to which others are seen as acting in prestigious ways; both on a 7-point scale from 1 (“not at all”) to 7 (“very much”). Each participant responded to all items of the Dominance and Prestige Scale twice; once referring to rich people, and once referring to poor people, both in the hypothetical society they had read about. We counterbalanced whether they rated rich or poor people first. To summarize, participants learned about the society either being high or low in inequality (between-subjects factor) and were then asked to rate the extent to which both rich and poor people (within-subjects factor) within that society are likely to be dominant and prestigious. The correlations among the variables are reported in S1 Table in S1 File.

### Results and discussion

First, with regards to participants’ expectations of dominance, to test whether participants expected rich people (*M* = 5.24, *SD* = 1.17, Cronbach’s α=0.86) to behave in more dominant ways than poor people (*M* = 3.50, *SD* = 0.98, Cronbach’s α=0.69), we collapsed across inequality condition and ran a mixed effects linear model with wealth as predictor and, because it was a within-subjects factor, we also included random intercepts for wealth. We used R packages lme4 [41] and lmerTest [42]. As hypothesized, participants expected rich people to act in more dominant ways than poor people across conditions, *β=*1.74, *p* < .001, 95%CI = [1.59, 1.89].

**Fig 1:**
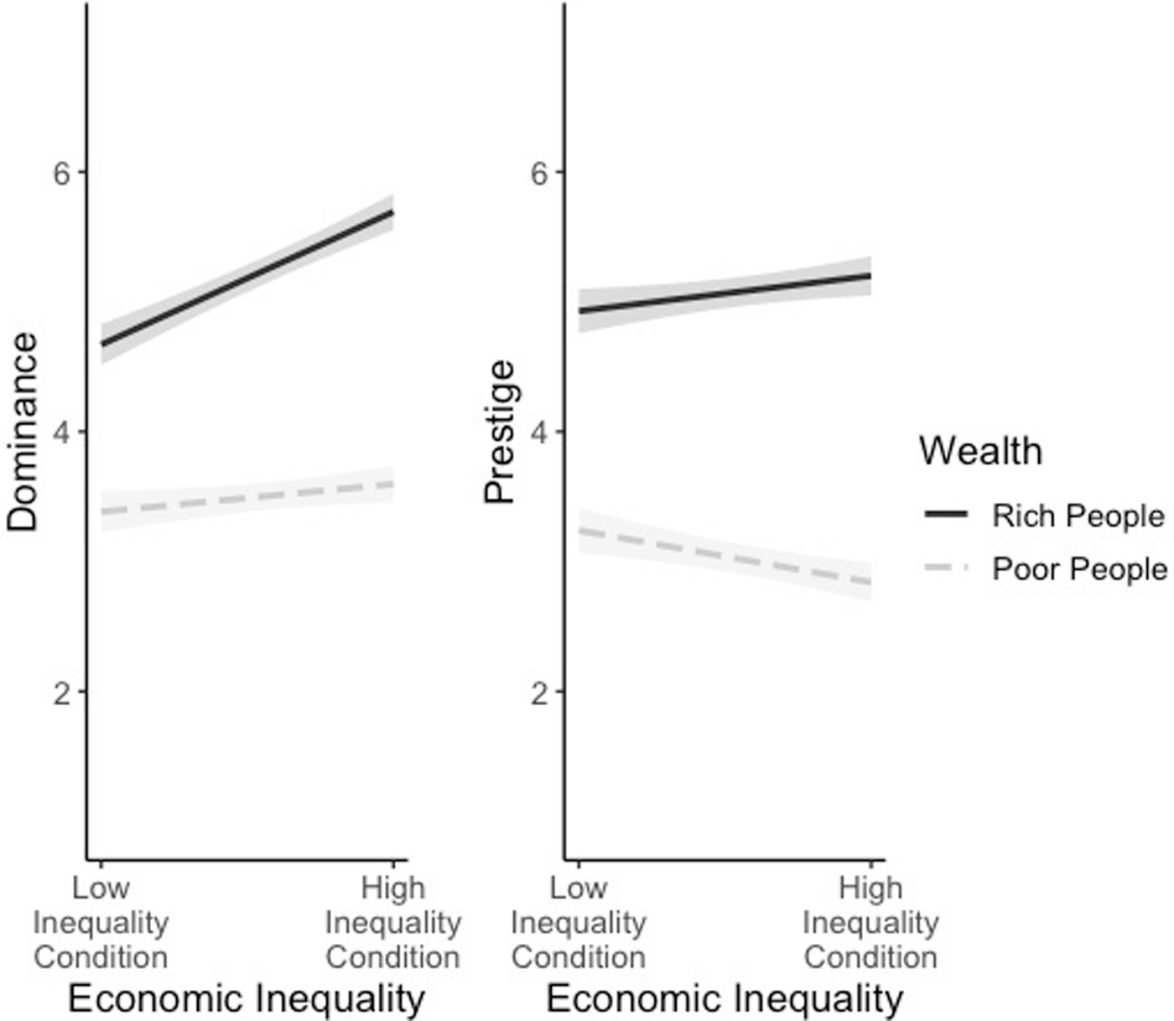
Association between economic inequality and expected dominance and prestige for rich and poor people. Intervals around regression lines are 95% confidence intervals. The dominance and prestige scales range from 1 (“not at all”) to 7 (“very much”).

To test the hypothesis that participants would expect rich people to behave in more dominant ways when living in a society of high (*n* = 206, M = 5.69, *SD* = 0.94) as opposed to low inequality (*n* = 162, *M* = 3.60, *SD* = 1.05), we ran a mixed linear effects model predicting dominance from inequality, wealth (rating rich and poor people), and the interaction between inequality and wealth; because wealth was a within-subjects factor, we included a random intercept for wealth. As hypothesized, a significant interaction emerged between inequality and wealth, *β=*0.81, *p* < .001, 95%CI = [0.51, 1.10]. Examining the simple slopes of inequality for the rich and poor conditions showed, consistent with our hypothesis, that inequality was associated with stronger perceptions of dominance among rich individuals *β=*1.02, *p < *.001, compared to poor, *β=*0.21, *p = *.046 (see Fig 1; we used R package interactions; Version 1.1.0; 43). As hypothesized, rich people were expected to be most dominant when living in a society of high inequality compared to one with low inequality; poor people were also expected to be more dominant in a highly unequal society than a more equal one, but this difference was much smaller.

Turning to the analyses with prestige, we tested whether participants expected rich people (*M* = 5.08, *SD* = 0.99) to behave in more (or less) prestigious ways than poor people (*M* = 3.02, *SD* = 1.23). We collapsed across inequality condition and ran a mixed effects linear model with wealth as predictor; because it was a within-subjects factor, we also included random intercepts for wealth. Participants expected rich people to act in more prestigious ways than poor people, *β=*2.06, *p* < .001, 95%CI = [1.90, 2.23].

We also tested whether participants would expect a difference in rich people’s tendency to behave prestigiously when living in a society of high (*M* = 5.20, *SD* = 0.96) as opposed to low inequality (*M* = 4.93, *SD* = 1.01). We ran a mixed linear effects model predicting prestige from inequality, wealth, and the interaction between inequality and wealth; because wealth was a within-subjects factor, we included a random intercept for wealth. A significant interaction emerged between inequality and wealth, *β=*0.67, *p* < .001, 95%CI = [0.35, 0.99]. Examining the simple slopes of inequality for the rich and poor conditions, we found that greater inequality was associated with greater perceptions of prestige among rich people, *β=*0.27, *p* = .020, and reduced perceptions of prestige among poor people, *β=*-0.40, *p* < .001. Thus, participants expected rich people in a society with high inequality to be more prestigious than rich people living in a society with low inequality while they expected poor people in a society with high inequality to be less prestigious than those in a society with low inequality.

To summarize, as hypothesized, participants expected people to be more dominant when they were described as rich as opposed to poor, and rich people were expected to be more dominant when living in a highly unequal society compared to a more equal society. Participants also expected rich people to be more prestigious, and poor people to be less prestigious, when living in a more unequal society.

## Study 1b

Study 1b was a conceptual replication of Study 1a. We tested the hypothesis that people would expect a store manager to lead their team in more dominant ways when the difference between the salary of the store manager and their employees was highly unequal compared to being less unequal. The focus of this study was on the difference between people of high SES in a context of high versus low inequality, and we therefore did not include conditions of low SES.

### Method

***Participants.*** We solicited a convenience sample of Americans on Prolific. Estimating an effect size of *d* = .30, 80% power, alpha of.05, and a two-tailed test, we needed a sample size of about 352 participants (calculated in GPower using the t test family and the Means difference between two independent means statistical test; 39). To ensure we were above this number, we collected data from 550 participants. After excluding participants who failed any of the three attention check questions (“How much larger is the salary of the store manager compared to a full-time sales person?”; “For this question, please choose the answer on the far right/at the bottom.”; “For any research question to be tested empirically, it is crucial that study participants take a study seriously and answer questions honestly. Please tell us whether you think we should analyze your results. You will be paid either way.”), the final sample consisted of 487 participants (*M* age =  32.52; 53% female, 46% male, 1% other; 62% Caucasian, 20% Asian, 8% Hispanic, 7% African American, 3% other).

#### Measures and stimuli.

***Economic inequality.*** Participants read a vignette about department stores where the difference in salary between the store managers and sales people vary (see below). In the high inequality condition, the store managers made 30 times as much as a full-time sales person, whereas in the low inequality condition the store managers made 3 times as much. In both cases, salaries were set by the HR department, to avoid inferences about dominance that might be made on the basis of a manager setting themselves a more disproportionate salary.

**Vignette**
For the different department stores around the country, the salaries for both the sales people and the stores managers are set by the HR department in the headquarters. Generally, the salaries for the different positions are the same across the different stores. The salary of a store manager is thirty times [three times] larger than the salary of a full time sales person in their store. An important aspect of a manager’s job is to use strategies to most effectively lead their team, in order to maximize their store’s performance.

***Dominance & Prestige.*** Participants completed a version of the Dominance and Prestige scale on a 7-point scale from 1 (“not at all”) to 7 (“very much”; 10) that was adapted to focus on managers’ perceived dominance and prestige with regards to their employees. Eight items assessed dominance (e.g., “The manager enjoys having control over their employees”; *M* = 4.54, *SD* = 0.87, Cronbach’s α =  0.76) and nine items assessed prestige (e.g., “Their employees respect and admire the manager”; *M* = 4.72, *SD* = 0.90, Cronbach’s α =  0.85; see SOM for list of the adapted items).

### Results and discussion

To test whether the manipulation was successful, participants were asked to report the extent of difference in pay between the store manager and their employees, on a scale from 1 (“not a big difference”) to 9 (extremely big difference”). Participants in the high inequality condition (*n* = 220, *M* = 8.34, *SD* = 1.04) perceived the pay difference to be significantly larger than participants in the low inequality condition (*n* = 267, *M* = 6.61, *SD* = 1.88), *β=*1.73, *p < *.001, 95%CI = [1.45, 2.00] [see S2 Table in S1 File for correlation between all variables; we used the R package stats for the regression, 37]. To test our hypothesis that participants would expect the store manager to behave more dominantly in the high compared to the low inequality condition, we regressed the mean dominance score on inequality condition. Consistent with the hypothesis, participants in the high inequality condition (*M* = 4.64, *SD* = 0.90) expected the store manager to be more dominant than did participants in the low inequality condition (*M* = 4.46, *SD* = 0.83), *β=*0.18, *p* = .020, 95%CI = [0.03, 0.34]. Thus, when participants were introduced to a more unequal context, they expected the wealthier manager to be more dominant towards the less wealthy employees, compared to when the income gap between the manager and the employees was smaller. Given the nature of this experiment’s design, we were unable to test for main effects of SES or an SES by inequality interaction.

Turning to the prestige analyses, we tested whether there would be a difference in expectations that the store manager would behave in prestigious ways between the high and low inequality conditions. There was no difference between participants in the high inequality condition (*M* = 4.70, *SD* = 0.93) and the low inequality condition (*M* = 4.74, *SD* = 0.87) in expecting the store manager to be prestigious, *β=*-0.34, *p* = .677, 95%CI = [-0.19, 0.13]. Hence, the results for prestige did not replicate the finding obtained in Study 1a.

In summary, Studies 1a and 1b focused on people’s expectations about the behavior of others who were of either high or low SES in a context where inequality was high versus low. In terms of both generalized perceptions about how wealthy and less wealthy people behave in a fictitious society and specific expectations for a store manager and their employees, participants reported greater expectations that wealthier people will engage in dominance-based strategies towards others when there is greater inequality compared to when there is less. Although the results were consistent in direction and statistical significance, the effect size was substantially larger in Study 1a than Study 1b, suggesting that some difference between the studies might produce a stronger association between economic inequality and expected dominance (e.g., inequality being determined by a third-party source in Study 1b). These subtle differences may be interesting to explore in future research. In contrast to the results with dominance, Studies 1a and 1b did not provide consistent findings with regards to people’s expectations about prestige.

## Study 2

In Study 2, we built on the findings of Studies 1a and 1b by focusing on people’s beliefs about their own behaviors. This study also extends the first two by moving beyond hypothetical scenarios to test whether our hypotheses hold for real people’s self-beliefs based on their actual SES and the inequality they perceive in their actual environment.

### Method

***Participants.*** We solicited a convenience sample of Americans on MTurk. The data were collected together with data for a different project. The sample size was based on that project and was determined to be 1040 participants. After excluding participants who failed one attention check question (“For this question, please choose the answer on the far left”) and/or who indicated that their answers shouldn’t be included in response to a question about whether they paid attention and took the study seriously, the final sample size was 962 participants (*M* age =  37.16; 53% female, 46% male; 72% Caucasian, 12% African American, 8% Hispanic, 6% Asian, 2% other). According to Schönbrodt and Perugini [40], a sample size of 470 participants allows us to detect a true correlation of.10 with a 95% CI when the corridor of stability is set to a half-width of.10. The sample size of this study is well beyond this number. Furthermore, an a priori power analysis for the interaction between subjective SES and inequality in G * Power [38], using the F family, the linear multiple regression (fixed model, R^2^ deviation from zero) statistical test, with an alpha level of 0.05, 80% power, 3 predictors, and an effect size of *f*^*2*^ = .02 for the interaction term [which is considered small; 43], shows that the needed minimum sample size for this analysis is 550 participants. Thus, the sample size for this study is larger than the minimum required sample size an a priori power analysis yielded for both statistical analyses.

***Social Class.*** We operationalized SES in two ways. First, participants reported their subjective SES [22] on a ladder with 10 rungs that indicated their relative standing in society (*M* = 5.29, *SD* = 1.74). Second, they indicated which of five social classes they thought they belonged to [i.e., poor, working-class, middle-class, upper middle-class, and upper-class; 44; *M* = 2.70, *SD* = 0.73].

***Perceived Economic Inequality & Unfairness Beliefs.*** Participants completed the 8-item Subjective Inequality Scale [26], which consists of two subscales: one measures how much inequality people perceive in their state of residence (*M* = 4.14, *SD* = 1.47, Cronbach’s α =  0.89; e.g., “Almost all of the money that is earned goes to only a few people”) and the other measures how unfair they find high levels of inequality (*M* = 4.86, *SD* = 1.40, α =  0.85; e.g., “It is extremely unfair if the overall amount of economic inequality is very high”) on a 7-point Likert scale from “strongly disagree” to “strongly agree”.

***Dominance & Prestige.*** Participants completed the 17-item Dominance and Prestige Scale [10; sample item for dominance “I enjoy having control over others, 8 items assess dominance, *M*=2.96, *SD*=1.18, α = 0.84; sample item for prestige “Members of my group respect and admire me”, 9 items assess prestige, *M* = 4.80, *SD* = 0.95, α =  0.79) on a 7-point Likert scale from 1 (“not at all”) to 7 (“very much”)].

### Results and discussion

We first tested the zero-order correlations between subjective SES (i.e., the ladder measure) as well as self-reported social class and self-reported dominance, and perceived inequality (using the inequality subscale of the Subjective Inequality Scale) and self-reported dominance. We used R package sjPlot [Version 2.8.11; 45]. As hypothesized, those of higher SES, operationalized with the ladder measure (*b* = 0.35, *p* < .001, 95%CI = [0.28, 0.42]) and with the social class categories (*b* = 0.28, *p* < .001, 95%CI = [0.20, 0.35]), described themselves as behaving in more dominant ways in comparison to those lower in SES and social class (note that all continuous predictor variables are standardized; see S3 Table in S1 File for correlations between all variables). Those who perceived greater inequality (*b* = 0.18, *p* < .001, 95%CI = [0.11, 0.25]) also described themselves as more dominant, across SES and class. Given that perceived inequality has been found to be associated with age, SES, and political orientation [26], and that dominance was also correlated with all three variables in the present study, we next reran all analyses including these covariates. The results held and are reported in S4 Table in S1 File.

**Fig 2:**
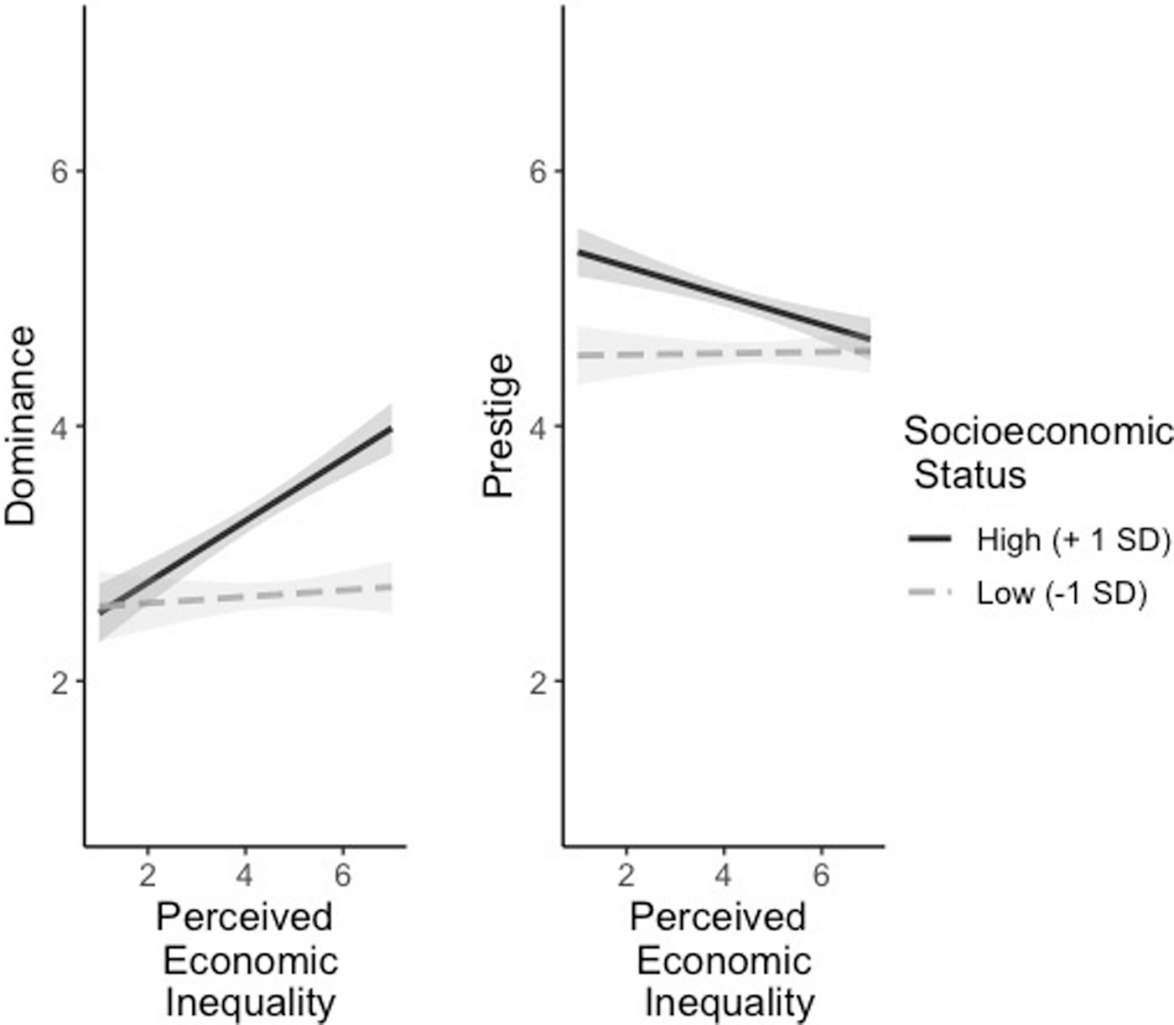
Association between perceived economic inequality and self-reported dominance and prestige for different levels of SES. Intervals around regression lines are 95% confidence intervals. The dominance and prestige scales range from 1 (“not at all”) to 7 (“very much”).

To test the hypothesis that people of high SES would report being more dominant when they perceived high as opposed to low levels of inequality in their environment, we regressed perceived amount of inequality, subjective SES, and the interaction term on self-reported dominance. We used R package stats for the interaction [37]. There was a significant interaction, *β=*0.16, *p* < .001, 95%CI = [0.09, 0.23], so we probed the simple slopes at 1 SD above and below the mean of subjective SES [Fig 2; we used R package interactions; 46]. Consistent with our hypothesis, at 1 SD above mean SES, the association between perceived economic inequality and self-reported dominance was *β=*0.35, *p < *.001, whereas at 1 SD below the mean, it was *β=*0.04, *p = *.483. Thus, when people of high SES perceive a great deal of inequality, they report being more dominant compared to when they perceive less inequality.

To test the robustness of these effects, we conducted the same analyses with the alternative 5-point measure of social class. There was again a significant interaction, *β=*0.11, *p* = .001, 95%CI = [0.05, 0.18], so we again probed for simple slopes at 1 SD above and below the mean of social class. Replicating the results using the ladder measure, at 1 SD above mean SES, the association between perceived economic inequality and self-reported dominance was *β=*0.32, *p < *.001, whereas at 1 SD below, it was *β=*0.10, *p = *.073. Both the interaction between SES and perceived inequality and between social class and perceived inequality held when controlling for age and political orientation (see S4 Table in S1 File). In summary, when people of a higher social class perceive a great deal of inequality, they report being more dominant compared to when they perceive less inequality. This pattern replicates what was observed in Studies 1a and 1b.

Turning to the prestige analyses, we tested the zero-order correlations between subjective SES, self-reported social class, perceived inequality, and self-reported prestige. People of higher SES (*b = *0.19, *p* < .001, 95%CI = [0.14, 0.25]) and of higher social class (*b = *0.17, *p* < .001, 95%CI = [0.11, 0.23]) described themselves as using more prestige strategies, and people who perceived more inequality (*b = *-0.11, *p* < .001, 95%CI = [-0.17, -0.05]) described themselves as using fewer prestige strategies. We controlled for age, SES, and political orientation. The results hold with these covariates and are reported in S5 Table in S1 File. We also tested for an interaction between perceived inequality and SES in predicting prestige strategies. There was a significant interaction, *β=*-0.09, *p* < .001, 95%CI = [-0.15, -0.03]. Since the interaction was significant, we probed for the simple slopes at 1 SD above and below the mean of subjective SES. At 1 SD above the mean the association between perceived economic inequality and self-reported prestige was *β=*-0.17, *p < *.001, whereas at 1 SD below the mean, it was *β=*0.01, *p = *.856. Thus, when people of high SES perceive a lot of inequality, they report having less prestige than when they perceive less inequality. This is the opposite pattern of results as was obtained in Study 1a.

To test the robustness of these effects, we conducted the same analyses with the alternative 5-point measure of social class. There was a marginally significant interaction, *β=*-0.06, *p* = .053, 95%CI = [-0.11, 0.0008]. Since the interaction was marginally significant, we probed again for the simple slopes at 1 SD above and below the mean of subjective SES. Replicating the results from above, at 1 SD above the mean the association between perceived economic inequality and self-reported prestige was *β=*-0.14, *p* = .001, whereas at 1 SD below the mean, it was *β=*-0.03, *p = *.523. Both the interaction between SES and perceived inequality and between social class and perceived inequality (it is marginally significant in the latter case) hold when controlling for age and political orientation (see S5 Table in S1 File).

## Study 3

In Study 2, we examined how the relation between self-reported SES and perceptions of inequality related to people’s expectations that they would behave dominantly. In Study 3, we manipulated both perceived SES and perceived inequality within a fictional society, and tested whether perceiving inequality to be high would increase people’s expectations of dominance. As in Study 1b, we focused only on people of high SES.

### Method

***Participants.*** We solicited a convenience sample of Americans on Prolific. Even though the analysis plan for this study was the same as in Study 1b, we estimated a smaller effect size here, as participants were asked to report the likelihood that they would behave dominantly, and not whether others would do so. Given that individuals tend to hold positive self-illusions [47], we expected it to be more difficult to elicit self-perceptions of dominance (compared to other perceptions) even under the predicted conditions, and thus expected a weaker effect. Estimating an effect size of *d* = .20, 80% power, alpha of.05, and a two-tailed test, we needed a sample size of about 788 participants [calculated in GPower using the t test family and the Means difference between two independent means statistical test; 39]. To ensure we were above this number, we collected data from 1113 participants. After excluding participants who failed at least one of two attention check questions (“Which income level have you been assigned to?”; “For this question, please move the slider to the very right”) and/or chose the wrong answer to a question testing basic English comprehension (we included this question because at the time of data collection, researchers were concerned about people from non-English speaking countries potentially accessing studies and creating noise in responses) and/or who indicated that their answers shouldn’t be included in response to a question about whether they paid attention and took the study seriously (due to an error, the second attention check question and the question testing English comprehension were only given to half of the sample), the final sample size was 922 participants (*M* age =  34.57; 52% female, 46% male, 1% other; 69% Caucasian, 14% Asian, 7% Hispanic, 6% African American, 4% other).

***Economic Inequality.*** Participants read about a fictitious society, “Bimboola”, that they had to imagine becoming part of [adapted from 47]. They learned that Bimboola has different income tiers, and that they would be assigned to one of these incomes. In the high inequality condition, participants learned that people in income group 1 earn, on average, 120,000 Bimboolean Dollars (BD) and in income group 2 they earn 20,000 BD. In the low inequality condition, participants learned that people in income group 1 earn, on average, 120,000 BD and in income group 2 they earn 90,000 BD. All participants were assigned to income group 1. Thus, across both conditions, participants had the same income, but the heterogeneity in income was higher in the high as opposed to the low inequality condition.

To strengthen the manipulation, participants were asked to choose among a house, car, and vacation spot for their life as a Bimboolean citizen. They saw three different options of houses, cars, and vacations that income group 1 could choose from and three different options that income group 2 could choose from. Importantly, across both conditions, the options provided for income group 1 were identical, and, because all participants were assigned to income group 1, they all had the same options. Thus, there was no difference in the kind of lifestyle they could afford. But the options they saw available to people in income group 2 differed by condition: In the high inequality condition, the difference in the value of the houses, cars, and vacation types between income group 1 and 2 were much more extreme than in the low inequality condition.

***Dominance & Prestige.*** To assess people’s expectations about their likelihood of behaving in dominant ways, they first read the following information about their job in Bimboola:

You are the CEO of a Bimboolean corporation. Both your salary and the salary of your employees were determined by the board and are beyond your control. As the CEO, your annual salary is 120,000 Bimboolean Dollars (BD) per year; a full time worker in the corporation has an average salary of BD 90,000 [20,000] per year.

Participants also learned that about 5% of all Bimboolean citizens belonged to income group 1 and 95% to income group 2.

***Open Response.*** To assess participants’ expectations of behaving in dominant ways, they were given three minutes to describe how they would lead people in their company. They were given the following prompt:

In the space below, please describe your management style. What strategies might you use to get your employees to do what you want them to do, in order to achieve your goals as CEO in Bimboola?
For example, how would you get your employees to work hard? How would you deal with conflicts between employees? How would you deal with an employee not fulfilling their responsibilities?
You will be able to proceed to the next question after 3 minutes.

***Dominance & Prestige Scale.*** After completing the open response question, participants completed a version of the Dominance and Prestige scale that was adapted for this situation on a 7-point scale from 1 (“not at all”) to 7 (“very much”;10). Six items assessed dominance (e.g., “I would enjoy having control over my employees”; *M* = 3.02, *SD* = 1.32, Cronbach’s α =  0.88), and six items assessed prestige (e.g., “I would try to make sure my employees respect and admire me”; *M* = 5.67, *SD* = 0.92, Cronbach’s α =  0.75; see SOM for the list of adapted items). We report the correlations among the variables in S6 Table in S1 File.

***Results and Discussion.*** To test whether the manipulation was successful, participants completed one item assessing the extent to which they found Bimboola to be equal or unequal on a 9-point scale from 1 (“extremely equal”) to 9 (“extremely unequal”). We used R package stats for the regression [37]. Participants in the high inequality condition (*n* = 430, *M* = 7.94, *SD* = 2.11) perceived Bimboola to be significantly more unequal than participants in the low inequality condition (*n* = 492, *M* = 3.76, *SD* = 2.12), *β=*4.18, *p* < .001, 95%CI = [3.90, 4.45]).

Next, we tested the hypothesis that people in the high inequality condition would expect to behave in more dominant ways than participants in the low inequality condition (note that all participants were assigned to be in the high SES, or CEO, condition). We tested this hypothesis in two ways. First, coders who were blind to the hypothesis and condition rated participants’ open-ended responses for the extent to which they described dominant behavior. Four research assistants (RAs) were trained together on the meaning of dominance and prestige and how to identify these themes in participants’ responses, and each response was coded by at least two of these RAs (interrater reliability: ICC =  0.79, 95% CI = [0.77, 0.81]; we calculated the ICC using the psych package and icc function in R based on the average rating across coders and coders’ agreement in a one-way random effects model which treats the coders but not the participants as random effects; an ICC between 0.75 and 0.90 is considered good; 48; note that it is not possible to use the two-way random-effects model (which treats participants as random effects as well) when each coder rates a different subset of participants [48].). An example response that was rated as not describing dominant behavior is: “I feel that in such a situation, there needs to be cooperation and respect for each other. Money differences shouldn’t make a difference in who is a leader and who is a worker. It makes sense to have everyone feel that they are worthy and are just as important as the other. If there are some incentives a leader can provide their employees like quality time with their family or friends, that may show they are valued.” An example response that was rated as describing dominant behavior is: “Showing authority and a sense of power is important in asserting dominance so I will be super confident and act like I can control them even though I don’t hold the power to determine their salary.”

Consistent with our hypothesis, participants in the high inequality condition (*M* = 2.03, *SD* = 1.10) described their expected management style as significantly more dominant than participants in the low inequality condition (*M* = 1.89, *SD* = 0.96), *β=*0.14, *p* = .037, 95%CI = [0.01, 0.28]).

Second, we compared ratings on the dominance scale, and found that participants in the high inequality condition (*M* = 3.12, *SD* = 1.40) indicated that they would behave in more dominant ways than participants in the low inequality condition (*M* = 2.93, *SD* = 1.24), *β=*0.19, *p* = .032, 95%CI = [0.02, 0.36]. Together, these results suggest that people of high SES expect themselves to behave more dominantly as leaders when they perceive their environment to be high as opposed to low in inequality.

Turning to the prestige analyses, we tested whether there was a difference in expecting to behave in prestigious ways for people of high SES in the high as opposed to the low inequality condition. We tested this in two ways. The same coders (as for dominance) also rated the extent to which the same open responses described prestigious behavior (interrater reliability: ICC =  0.80, 95% CI = [0.78, 0.82]). Participants in the high inequality condition (*M* = 3.02, *SD* = 1.17) described their management style as marginally less prestigious than participants in the low inequality condition (*M* = 3.16, *SD* = 1.13), *β=*-0.14, *p* = .072, 95%CI = [-0.29, 0.01].

Second, there was no difference between participants in the high inequality condition (*M* = 3.12, *SD* = 1.40) and participants in the low inequality condition (*M* = 2.93, *SD* = 1.24) in indicating in response to the prestige items that they would behave in more prestigious ways, *β=*0.03, *p* = .624, 95%CI = [-0.09, 0.15].

## General discussion

Across four studies, we found that people expect others and themselves to behave more dominantly if they are of high as opposed to low SES (although we tested this particular effect in only two of the four studies), and that these expectations of dominance for people of high SES are greatest when economic inequality is high as opposed to low. These findings suggest that perceiving high levels of inequality exacerbates the effects of SES on adopting a dominance strategy. In contrast, there were no consistent effects of inequality on the tendency to adopt a prestige strategy.

People of higher SES have more power, control over their lives, and need to rely less on others to get their way [e.g., 1,16]. As a result, they may also be more willing to use dominant tactics to get their way [18], and hence to expect that others of their social class, as well as themselves, would use dominant tactics to get ahead. In line with this thinking, research finds that people of higher SES tend to feel more entitled and narcissistic [7].

If people of higher SES say they would behave in more dominant ways (and expect others to behave in more dominant ways when they are of high as opposed to low SES) because they have greater access to valued resources and hence, feel more powerful, then there should be an interaction between SES and inequality perceptions such that people of high SES become even more dominant when they perceive inequality to be high. We found evidence for this expectation across all four studies – two studies focused on people’s expectations of other people’s behavior and two focused on expectations about people’s own behavior (note that in two experiments everyone was assigned to be high SES (Study 3) or think of another person of high SES (Study 1b) and the only variable manipulated was inequality).

The observed main effect of SES on expected dominance replicates past research showing that people say they are more likely to behave in dominant ways when they are of high as opposed to low SES (Study 2, [18]), and further shows that people also expect others to behave in more dominant ways when they are of high as opposed to low SES (Study 1b). We extended this prior research by finding that the amount of inequality people perceive in society moderates the extent to which people of high SES (both others and themselves) are expected to behave dominantly. These results suggest that high SES does not lead unequivocally to the same expectations of dominant behavior, but that context matters. Specifically, these findings suggest that the effects of SES on dominance strategies are exacerbated under conditions of high perceived economic inequality. This finding is important for at least two reasons. First, it suggests that attaining a complete understanding of the psychological effects of SES may not be possible without also considering the effects of perceived inequality. Second, it suggests that at least some of the ill effects that have been observed to be associated with being of high SES may not be inherent high SES per se, but rather to being high SES in a context of high perceived inequality.

In addition, our finding of greater expectations of dominance for oneself and others suggest that dominance might be evaluated as more appropriate and desirable by individuals living in societies with high inequality. Dispositional factors can affect evaluations of the effectiveness and benevolence of dominant behavior [49], and the present research raises the possibility that economic inequality might be a situational factor that exerts similar effects. Future research might build on these findings by examining the perceived value of dominance in societies that vary in economic inequality.

These studies also have several limitations. First, all studies focused exclusively on people’s expectations about other people’s (Studies 1a and 1b) or their own (Studies 2 and 3) behavior. To the extent that it is adaptive to tailor the employment of a dominance strategy to one’s own SES and the amount of perceived inequality in the environment, it would also seem adaptive to tailor one’s expectations about the likelihood that others and oneself will behave dominantly. However, all of our studies used self-report measures to assess expected levels of dominance and prestige, which are subject to self-serving biases such as social desirability bias, and we did not measure any actual behaviors, making this an important direction for future research. While people may expect themselves and others to act more dominantly in highly unequal contexts, an important factor may also be how inequality *and one’s position in the social hierarchy*, may have come about. For instance, perhaps people of high SES behave differently when they grew up being of high SES as opposed to climbing the social hierarchy as an adult. On this note, future studies would benefit from investigating how the degree of actual, or perceived, economic mobility might influence these effects because the capacity for economic mobility helps determine the expected effectiveness of any rank attainment or maintenance strategy. It also remains an open question to what extent cultural models about wealth and dominance affect people’s actual behavior.

Furthermore, we focused only on *perceptions* of economic inequality and SES, rather than objective indicators of these variables. Though perceptions of these economic factors are associated with various self-centered cognitions and behaviors [e.g., 7,18,50], and it seems plausible that perceiving these factors (in the environment) is itself adaptive, future research would benefit from testing how these results compare to research examining objective indices of inequality and SES.

In addition, and in line with previous research, we argued that the effects of SES are mediated through a heightened sense of power and greater self-reliance among individuals of higher SES. However, we did not assess these proposed mediators, and it is possible that different mechanisms might better (or additionally) explain the effects we found.

Finally, all studies were conducted exclusively with online and American samples, which limits their broader generalizability. In particular, economic inequality in the US is higher than in most other industrialized countries, and it is an open question whether we would find the same results in less unequal countries. Additionally, in countries where poverty rates are high, inequality may be less relevant to people’s psychology and behavior. People might be more affected by their absolute material conditions (especially if their livelihood is threatened) than by their relative position in society, and if absolute material conditions are quite scarce over time, dominance might no longer be a rational strategy for anyone, regardless of SES [19]. Furthermore, the effects of SES on various self-centered cognitions and behaviors may themselves depend on what it means to be of high SES in a given society. In societies that embrace norms of *noblesse oblige* and view those with high SES as having a responsibility to help the less privileged, higher SES may well be associated with less self-centeredness, and consequently perhaps less dominance. Future research would benefit from testing these associations in different contexts to identify potential contextual variability.

Additionally, in places with less anonymity, people of higher SES might also show fewer self-centered cognitions and behaviors and become more dependent on attaining prestige and behaving in ways that benefit others. Furthermore, if status is based on other aspects than material wealth, such as a person’s occupational prestige or adherence to religious norms [51], self-centered cognitions and behaviors may be less likely to occur among high SES individuals, or may occur in different contexts.

To conclude, we find evidence that people expect others and themselves to act more dominantly when they are of higher SES, replicating previous research. But the extent to which people of high SES are expected to be more dominant depends on contextual factors: the more economic inequality people perceive to be in their environment, the more dominant behavior they expect. This work showcases the importance of considering psychological effects of SES alongside those of inequality, because they are inevitably intertwined. Without considering the socioeconomic structure of a society, we lose important information about the meaning of individuals’ SES and the downstream consequences of their social position.

## Supporting information

S1 File **Table S1.** Correlation between all Between-Subjects Variables in Study 1a. **Table S2.** Correlation between All Variables in Study 1b. **Table S3**. Correlation between All Variables in Study 2. Table S4 Relationship between 1) perceived economic inequality, 2) SES and 3) an interaction between inequality and SES in predicting dominance with covariates in Study 2. **Table S5.** Relationship between 1) perceived economic inequality, 2) SES and 3) an interaction between inequality and SES in predicting prestige with covariates in Study 2. **Table S6.** Correlation between all Variables in Study 3.
(DOCX)
