# Supplementary material for: When all is unequal, the rich get dominant: Inequality leads to expectations of dominant leadership among those high in SES
Source: PLoS One. 2025 Apr 9;20(4):e0321138. doi: 10.1371/journal.pone.0321138 (PMC11981125; doi:10.1371/journal.pone.0321138)
Supplement: S1 File — Table S1. Correlation between all Between-Subjects Variables in Study 1a. Table S2. Correlation between All Variables in Study 1b. Table S3. Correlation between All Variables in Study 2. Table S4 Relationship between 1) perceived economic inequality, 2) SES and 3) an interaction between inequality and SES in predicting dominance with covariates in Study 2. Table S5. Relationship between 1) perceived economic inequality, 2) SES and 3) an interaction between inequality and SES in predicting prestige with covariates in Study 2. Table S6. Correlation between all Variables in Study 3. (DOCX) [file pone.0321138.s001.docx]

**Supplementary Material for**

**Perceiving high economic inequality causes people to expect more dominance,**

especially from people of high social class

Study 1a

Table S1 Correlation between all Between-Subjects Variables in Study 1a.

|  | *Dominance* | *Prestige* | *Economic Inequality* | *SES* | *Social Class* | *Conservatism* | *Age* |
| --- | --- | --- | --- | --- | --- | --- | --- |
| *Dominance* |  |  |  |  |  |  |  |
| *Prestige* | 0.182 *(<.001)* |  |  |  |  |  |  |
| *Economic Inequality* | 0.396 *(<.001)* | -0.055 *(.298)* |  |  |  |  |  |
| *SES* | 0.113 *(.031)* | 0.180 *(.001)* | -0.018 *(.734)* |  |  |  |  |
| *Social Class* | 0.108 *(.039)* | 0.155 *(.003)* | -0.014 *(.790)* | 0.765 *(<.001)* |  |  |  |
| *Conservatism* | -0.146 *(.005)* | 0.051 *(.335)* | -0.117 *(.026)* | 0.044 *(.402)* | -0.016 *(.761)* |  |  |
| *Age* | -0.024 *(.652)* | 0.020 *(.707)* | -0.071 *(.180)* | 0.153 *(.004)* | 0.116 *(.027)* | 0.163 *(.002)* |  |
| *Gender* | 0.058 *(.269)* | 0.061 *(.246)* | -0.115 *(.029)* | 0.137 *(.009)* | 0.102 *(.052)* | 0.145 *(.006)* | 0.018 *(.730)* |
| *Computed correlation used pearson-method with listwise-deletion.* | | | | | | | |

*Notes.* Economic Inequality: 0 = low inequality condition, 1 = high inequality condition. Gender: 0 = female, 1 = male. Conservatism: 1= very liberal, 7 = very conservative (mean score of political orientation on economic and social issues, *M*=3.38, *SD*=1.50). Dominance and prestige scores are collapsed across SES conditions.

Study 1b

**Adapted Items of the Dominance & Prestige Scale (Cheng et al., 2010)**

**Dominance Items**

1. “The manager enjoys having control over their employees”
2. “The manager often tries to get their own way regardless of what their employees may want”
3. “The manager is willing to use aggressive tactics to get their way”
4. “The manager tries to control their employees rather than being controlled by them”
5. “The manager does not have a forceful or dominant personality” (reverse-scored)
6. “The manager's employees know it is better to let them have their way”
7. “The manager does not enjoy having authority over their employees”
8. “Some of their employees are afraid of the manager” (reverse-scored)

**Prestige Items**

1. “Their employees respect and admire the manager”
2. “Their employees do not want to be like the manager” (reverse-scored)
3. “Their employees always expect the manager to be successful”
4. “Their employees do not value the manager's opinion” (reverse-scored)
5. “The manager is held in high esteem by their employees”
6. “The manager's unique talents and abilities are recognized by their employees”
7. “The manager is considered an expert on some matters by their employees”
8. “Their employees seek the manager's advice on a variety of matters”
9. “Their employees do not enjoy hanging out with the manager” (reverse-scored)

Table S2 Correlation between All Variables in Study 1b.

|  | Dominance | Prestige | Economic  Inequality | Manipulation  Check | SES | Social  Class | Conservatism | Age |
| --- | --- | --- | --- | --- | --- | --- | --- | --- |
| Dominance |  |  |  |  |  |  |  |  |
| Prestige | -0.246 (<.001) |  |  |  |  |  |  |  |
| Economic  Inequality | 0.101 (.026) | -0.018 (.692) |  |  |  |  |  |  |
| Manipulation  Check  (higher score  = perceived as  more unequal) | 0.204 (<.001) | -0.014 (.754) | 0.483 (<.001) |  |  |  |  |  |
| SES | 0.005 (.904) | 0.040 (.379) | -0.082 (.070) | -0.054 (.238) |  |  |  |  |
| Social Class | -0.023 (.620) | 0.049 (.279) | -0.122 (.007) | -0.082 (.072) | 0.719 (<.001) |  |  |  |
| Conservatism | -0.105 (.021) | 0.108 (.017) | -0.015 (.734) | -0.042 (.352) | 0.090 (.047) | 0.106 (.019) |  |  |
| Age | 0.044 (.330) | 0.002 (.957) | 0.010 (.833) | 0.036 (.432) | 0.060 (.190) | -0.011 (.809) | 0.137 (.002) |  |
| Gender | 0.054 (.241) | -0.014 (.762) | -0.019 (.676) | 0.013 (.770) | 0.056 (.218) | 0.164 (<.001) | 0.151 (.001) | -0.063 (.165) |
| Computed correlation used pearson-method with listwise-deletion. | | | | | | | | |

*Note.* Economic Inequality: 0 = low inequality condition, 1 = high inequality condition. Gender: 0 = female, 1 = male. Conservatism: 1= very liberal, 7 = very conservative (mean score of political orientation on economic and social issues, *M*=3.21, *SD*=1.45).

Study 2

Table S3 Correlation between All Variables in Study 2.

|  | *Dominance* | *Prestige* | *Perceived*  *Inequality* | *Unfairness*  *Judgments of*  *Inequality* | *SES* | *Social*  *Class* | *Conservatism* | *Age* |
| --- | --- | --- | --- | --- | --- | --- | --- | --- |
| *Dominance* |  |  |  |  |  |  |  |  |
| *Prestige* | 0.128 *(<.001)* |  |  |  |  |  |  |  |
| *Perceived*  *Inequality* | 0.151 *(<.001)* | -0.114 *(<.001)* |  |  |  |  |  |  |
| *Unfairness*  *Judgments*  *of Inequality* | -0.050 *(.120)* | -0.016 *(.615)* | 0.591 *(<.001)* |  |  |  |  |  |
| *SES* | 0.299 *(<.001)* | 0.203 *(<.001)* | -0.097 *(.003)* | -0.100 *(.002)* |  |  |  |  |
| *Social*  *Class* | 0.235 *(<.001)* | 0.176 *(<.001)* | -0.129 *(<.001)* | -0.112 *(.001)* | 0.754 *(<.001)* |  |  |  |
| *Conservatism* | 0.215 *(<.001)* | 0.004 *(.901)* | -0.228 *(<.001)* | -0.418 *(<.001)* | 0.241 *(<.001)* | 0.180 *(<.001)* |  |  |
| *Age* | -0.159 *(<.001)* | -0.026 *(.428)* | -0.170 *(<.001)* | -0.125 *(<.001)* | -0.027 *(.412)* | -0.020 *(.537)* | 0.107 *(.001)* |  |
| *Gender* | 0.220 *(<.001)* | 0.029 *(.371)* | 0.065 *(.046)* | -0.017 *(.600)* | 0.083 *(.010)* | 0.077 *(.018)* | 0.097 *(.003)* | -0.106 *(.001)* |
| *Computed correlation used pearson-method with listwise-deletion.* | | | | | | | | |

*Note.* Gender coded as 1 = male, 0 = female. Conservatism: 1= very liberal, 7 = very conservative (mean score of political orientation on economic and social issues, *M*=3.84, *SD*=1.74). Dominance and prestige scores are collapsed across SES conditions.

Table S4 Relationship between ****1) perceived economic inequality, 2) SES and 3) an interaction between inequality and SES in predicting dominance with covariates in Study 2.****

|  | **Dominance** | | | **Dominance** | | | **Dominance** | | | **Dominance** | | |
| --- | --- | --- | --- | --- | --- | --- | --- | --- | --- | --- | --- | --- |
| *Predictors* | *Estimates* | *CI* | *p* | *Estimates* | *CI* | *p* | *Estimates* | *CI* | *p* | *Estimates* | *CI* | *p* |
| (Intercept) | 2.96 | 2.89 – 3.03 | **<0.001** | 2.96 | 2.89 – 3.03 | **<0.001** | 2.97 | 2.91 – 3.04 | **<0.001** | 2.97 | 2.90 – 3.04 | **<0.001** |
| SES | 0.31 | 0.24 – 0.38 | **<0.001** |  |  |  | 0.27 | 0.19 – 0.34 | **<0.001** |  |  |  |
| Perceived Inequality | 0.24 | 0.17 – 0.31 | **<0.001** | 0.25 | 0.18 – 0.32 | **<0.001** | 0.22 | 0.15 – 0.29 | **<0.001** | 0.24 | 0.17 – 0.31 | **<0.001** |
| Conservatism | 0.25 | 0.18 – 0.32 | **<0.001** | 0.28 | 0.21 – 0.36 | **<0.001** | 0.23 | 0.16 – 0.30 | **<0.001** | 0.27 | 0.20 – 0.35 | **<0.001** |
| Age | -0.16 | -0.23 – -0.09 | **<0.001** | -0.17 | -0.24 – -0.10 | **<0.001** | -0.16 | -0.23 – -0.10 | **<0.001** | -0.17 | -0.24 – -0.10 | **<0.001** |
| Social Class |  |  |  | 0.26 | 0.18 – 0.33 | **<0.001** |  |  |  | 0.24 | 0.17 – 0.31 | **<0.001** |
| Perceived Inequality * SES |  |  |  |  |  |  | 0.13 | 0.06 – 0.19 | **<0.001** |  |  |  |
| Perceived Inequality * Social Class |  |  |  |  |  |  |  |  |  | 0.09 | 0.02 – 0.16 | **0.009** |
| Observations | 962 | | | 962 | | | 962 | | | 962 | | |
| R^2^ / R^2^ adjusted | 0.176 / 0.173 | | | 0.156 / 0.153 | | | 0.188 / 0.183 | | | 0.162 / 0.158 | | |

*Note.* Conservatism: 1= very liberal, 7 = very conservative (mean score of political orientation on economic and social issues, *M*=3.84, *SD*=1.74). Age, conservatism, perceived inequality, SES, and social class are standardized.

Table S5 Relationship between **1) perceived economic inequality, 2) SES and 3) an interaction between inequality and SES in predicting prestige with covariates in Study 2.**

|  | **Prestige** | | | **Prestige** | | | **Prestige** | | | **Prestige** | | |
| --- | --- | --- | --- | --- | --- | --- | --- | --- | --- | --- | --- | --- |
| *Predictors* | *Estimates* | *CI* | *p* | *Estimates* | *CI* | *p* | *Estimates* | *CI* | *p* | *Estimates* | *CI* | *p* |
| (Intercept) | 4.80 | 4.74 – 4.86 | **<0.001** | 4.80 | 4.74 – 4.86 | **<0.001** | 4.79 | 4.73 – 4.85 | **<0.001** | 4.79 | 4.73 – 4.85 | **<0.001** |
| SES | 0.20 | 0.14 – 0.26 | **<0.001** |  |  |  | 0.22 | 0.16 – 0.29 | **<0.001** |  |  |  |
| Perceived Inequality | -0.11 | -0.17 – -0.05 | **<0.001** | -0.10 | -0.17 – -0.04 | **0.001** | -0.10 | -0.16 – -0.03 | **0.002** | -0.10 | -0.16 – -0.04 | **0.002** |
| Conservatism | -0.07 | -0.13 – -0.00 | **0.037** | -0.05 | -0.11 – 0.02 | 0.146 | -0.05 | -0.11 – 0.01 | 0.104 | -0.04 | -0.10 – 0.02 | 0.210 |
| Age | -0.03 | -0.09 – 0.03 | 0.371 | -0.03 | -0.09 – 0.03 | 0.325 | -0.03 | -0.09 – 0.03 | 0.382 | -0.03 | -0.09 – 0.03 | 0.336 |
| Social Class |  |  |  | 0.16 | 0.10 – 0.22 | **<0.001** |  |  |  | 0.17 | 0.11 – 0.23 | **<0.001** |
| Perceived Inequality * SES |  |  |  |  |  |  | -0.08 | -0.14 – -0.02 | **0.008** |  |  |  |
| Perceived Inequality * Social Class |  |  |  |  |  |  |  |  |  | -0.05 | -0.11 – 0.01 | 0.078 |
| Observations | 962 | | | 962 | | | 962 | | | 962 | | |
| R^2^ / R^2^ adjusted | 0.057 / 0.053 | | | 0.043 / 0.039 | | | 0.064 / 0.059 | | | 0.046 / 0.041 | | |

*Note.* Conservatism: 1= very liberal, 7 = very conservative (mean score of political orientation on economic and social issues, *M*=3.84, *SD*=1.74). Age, conservatism, perceived inequality, SES, and social class are standardized.

**Adapted Version of the Dominance & Prestige Scale (Cheng et al., 2010)**

**Dominance Items**

1. “I would enjoy having control over my employees”
2. “I would often try to get my own way regardless of what my employees may want”
3. “I would be willing to use aggressive tactics to get my way”
4. “I would try to control my employees rather than permit them to control me”
5. “I would make sure my employees know it is better to let me have my way”

**Prestige Items**

1. “I would try to make sure my employees respect and admire me”
2. “I would try to make sure that my employees value my opinion”
3. “I would try to demonstrate my unique talents and abilities to my employees”
4. “I would want my employees to seek my advice on a variety of matters”
5. “I would want my employees to enjoy working with me”

Table S6 Correlation between all Variables in Study 3.

|  | *Dominance*  *(from Dominance*  *& Prestige Scale)* | *Dominance*  *(coded open response)* | *Prestige*  *(from Dominance*  *& Prestige Scale)* | *Prestige*  *(coded open response)* | *Economic Inequality* | *Manipulation Check*  *(higher score = more unequal)* | *SES* | *Social Class* | *Conservatism* | *Age* |
| --- | --- | --- | --- | --- | --- | --- | --- | --- | --- | --- |
| *Dominance*  *(from Dominance*  *& Prestige Scale)* |  |  |  |  |  |  |  |  |  |  |
| *Dominance*  *(coded open*  *response)* | 0.365 *(<.001)* |  |  |  |  |  |  |  |  |  |
| *Prestige*  *(from Dominance*  *& Prestige Scale* | 0.044 *(.191)* | -0.104 *(.002)* |  |  |  |  |  |  |  |  |
| *Prestige*  *(coded open*  *response)* | -0.393 *(<.001)* | -0.606 *(<.001)* | 0.149 *(<.001)* |  |  |  |  |  |  |  |
| *Economic*  *Inequality* | 0.070 *(.036)* | 0.069 *(.039)* | 0.002 *(.956)* | -0.062 *(.064)* |  |  |  |  |  |  |
| *Manipulation*  *Check*  *(higher score*  *= perceived to*  *be more unequa0* | 0.094 *(.005)* | 0.084 *(.012)* | 0.012 *(.721)* | -0.083 *(.013)* | 0.709 *(<.001)* |  |  |  |  |  |
| *SES* | 0.148 *(<.001)* | -0.024 *(.467)* | 0.027 *(.416)* | -0.031 *(.348)* | -0.013 *(.700)* | 0.014 *(.669)* |  |  |  |  |
| *Social Class* | 0.174 *(<.001)* | -0.044 *(.190)* | 0.025 *(.450)* | -0.029 *(.383)* | -0.008 *(.801)* | -0.001 *(.978)* | 0.744 *(<.001)* |  |  |  |
| *Conservatism* | 0.284 *(<.001)* | 0.112 *(.001)* | 0.026 *(.430)* | -0.184 *(<.001)* | -0.037 *(.264)* | 0.023 *(.495)* | 0.149 *(<.001)* | 0.133 *(<.001)* |  |  |
| *Age* | 0.011 *(.732)* | -0.102 *(.002)* | 0.014 *(.677)* | 0.006 *(.866)* | -0.036 *(.278)* | -0.001 *(.969)* | 0.059 *(.077)* | 0.029 *(.390)* | 0.188 *(<.001)* |  |
| *Gender* | 0.307 *(<.001)* | 0.166 *(<.001)* | -0.091 *(.007)* | -0.235 *(<.001)* | -0.027 *(.428)* | -0.045 *(.186)* | 0.092 *(.006)* | 0.128  *(<.001)* | 0.142 *(<.001)* | -0.062 *(.064)* |
| *Computed correlation used pearson-method with listwise-deletion.* | | | | | | | | | | |

***Note.*** Economic Inequality: 0 = low inequality condition, 1 = high inequality condition. Gender: 0 = female, 1 = male. Conservatism: 1= very liberal, 7 = very conservative (mean score of political orientation on economic and social issues, *M*=3.22, *SD*=1.66).
